# Effect of Isolated Obesity on Left Ventricular Function and Structure: A Single-Center Experience

**DOI:** 10.7759/cureus.13988

**Published:** 2021-03-19

**Authors:** Jamilah AlRahimi, Abdulbari Aboud, Abdullah S AlQuhaibi, Yazan Almaghrabi, Yousef S Alghamdi, Hani N Mufti

**Affiliations:** 1 Cardiology, King Faisal Cardiac Center, King Abdulaziz Medical City, Ministry of National Guard Health Affairs, King Abdullah International Medical Research Center, King Saud bin Abdulaziz University for Health Science, Jeddah, SAU; 2 College of Medicine, Ministry of National Guard Health Affairs, King Abdullah International Medical Research Center, King Saud bin Abdulaziz University for Health Science, Jeddah, SAU; 3 Pediatrics, Ministry of National Guard Health Affairs, King Abdullah International Medical Research Center, King Saud bin Abdulaziz University for Health Science, Jeddah, SAU; 4 Cardiac Surgery, King Faisal Cardiac Center, King Abdulaziz Medical City, Ministry of National Guard Health Affairs, King Abdullah International Medical Research Center, King Saud bin Abdulaziz University for Health Science, Jeddah, SAU

**Keywords:** heart hypertrophy, overweight, cardiac output, obesity, risk

## Abstract

Background and objectives

Obesity can increase cardiac mass and affect cardiac performance independently from other risk factors. Several studies have identified an association in patients who already have comorbidities, however, few studies focused on obesity as an isolated risk factor. This study aimed to assess the associations between isolated obesity and heart morphological and functional characteristics.

Methods

This was a cross-sectional study that recruited 114 patients referred for echocardiographic study in King Faisal Cardiac Center. Adult patients who had a body mass index (BMI) above 25 kg/m^2^ were included, while patients with comorbidities such as hypertension, diabetes mellitus, dyslipidemia, or those who use medications for chronic diseases were excluded from this study. Variables of interest that we collected were age, gender, weight, BMI, and those related to morphological and functional changes in the heart including left ventricular mass index (LVMI), LV end-diastolic volume, and left ventricular ejection fraction (LVEF).

Results

Most of the study participants (63.8%) were class II or class III obesity and about 80% were males. The mean ± SD of LVEF was 55.7% ± 2.8%, while the mean of the left ventricular mass index was 28.5±5.84. The mean of LV end-diastolic volume index (LVEDVI) was slightly higher among males than females (48.8±11.6 versus 46.4±11.7 ml/m^2^), however, this difference was not statistically significant (p-value= 0.395). There was no correlation between BMI and LVMI in females (R - 0.226, R^2^ 0.05, P-value 0.37), while the LVMI was found to have a negative correlation between BMI and male gender that was significant (R - 0.292, R^2^ 0.09, P-value 0.0052). It was found that there is no correlation between LVEF and BMI for males and females (male= R 0.093, R^2 ^0.032, P-value 0.093; female= R 0.172, R^2^ 0.029, P-value 0.434). With regards to the LVEDVI, there was a negative correlation between higher BMI and male gender that was significant (male= R - 0.396, R^2^ 0.157, P-value 0.0001) while it was not significant in females (R -0.0298, R^2^ 0.0009, P-value 0.893).

Conclusions

We have found that cardiac function is not affected by isolated obesity. However, indexed cardiac parameters like LVM and LV end diastolic volume were negatively correlated with higher BMI and positively correlated with relative wall thickness (RWT) only in males. This negative correlation might be one of the triggers to the development of obesity-induced cardiomyopathy.

## Introduction

The effect of obesity in the heart has been identified since 16th and 17th centuries when Senac and Corvisart noticed the fat surrounding heart in adipose people. They assumed that these adipose tissues can affect heart performance [1]. The first scientific study that assessed the association between obesity and heart function was published by Smith and Willius in 1933; there was a relationship between cardiac mass and obesity [2]. Recent literature linked obesity to elevated cardiac output (CO), left ventricular mass (LVM), ejection fraction (LVEF), and reduced peripheral vascular resistance (PVR) [1,3,4]. The infiltration of fatty cells in heart tissues is also noticed in obese persons and is as called “obesity-induced cardiomyopathy” [5].

In the last three decades, the prevalence of overweight or obese adults has increased from 28.8% to 36.9% in males, and from 30% to 38% in females [6]. In 2013, the prevalence of overweight or obesity in developed countries was 23.8% in boys and 22.6% in girls, while in developing countries it was 12.9% in boys and 13.4% in girls [6]. It is predicted that the prevalence of obesity in Saudi Arabia will increase from 22% to 41% in males and from 36% to 78% in females by the year 2022 [7]. This high prevalence can be associated with serious health problems, particularly heart disease. Furthermore, obesity can cause diastolic dysfunction, even in the absence of risk factors such as hypertension, coronary heart diseases, diabetes mellitus, and hyperlipidemia [8]. Super morbid obesity, body mass index (BMI) > 50 kg/m^2^, can increase cardiac mass and negatively affect heart performance independently from other risk factors. Many studies demonstrated this association among patients with other comorbidities [9-11], however, few studies focused on the isolated risk of obesity [8,12]. This study aimed to assess the associations between isolated obesity and heart morphological and functional characteristics.

## Materials and methods

This was a cross-sectional study in which all patients referred for echocardiographic study in King Faisal Cardiac Center during the period from January 2011 to December 2016 were included. Adult patients defined as overweight who had a BMI ≥ 25 kg/m^2^ and patients with obesity who had BMI ≥ 30 kg/m^2^ were included. The exclusion criteria were patients with comorbidities such as hypertension, diabetes mellitus, and dyslipidemia. Moreover, patients with cardiac diseases or those who use medications for chronic diseases were excluded from this study. The patient’s records were reviewed, and any records containing missing data were excluded. After that, the data from hospital records of 114 referred patients were recruited in this study. The independent variables for which data collected were age, gender, weight, and BMI. The dependent variables were those related to morphological and functional changes in the heart including LVM index (LVMI), LV end-diastolic volume, and LVEF. All data were coded for storage and analysis in the computer using Statistical Package of Social Sciences SPSS version 22 (IBM Corp., Armonk, NY). The descriptive statistics were calculated using frequencies, percentages, and frequency distribution tables, while inferential statistics such as t-test was used to detect significant differences between groups at less than 0.05 significance level. The relationship between BMI and continuous variables was assessed using Pearson's correlation to assess the direction and magnitude of correlation coefficient (r) effect of BMI on the other variable. The coefficient of determination (R2) was used to assess the strength of the relationship. The confidentiality of the personal data was ensured by using the anonymous data sheet in the analysis. The patients were informed about the study objectives and their freedom to participate without any enforcement or temptation, then a written consent was obtained from them. This study was approved by King Abdullah International Medical Research Center on October 15, 2017.

## Results

Out of 114 study participants, about 80% were males as the study was done in a National Guard Health Affair Facilities with a predominantly male population. The majority of the study participants (56.1%) aged 31-50 years old, the mean ± SD was (41.42±12.4) years old, ranging from 17 to 71 years old. The mean ± SD for age in males was 39.8±12 and females was 47.8±12.2 (p-value= <0.001). Most of the study participants had class II and class III obesity (both were 31.9%), while the overweight participants were only 12.4% and the class I participants were 23.7% (Table 1). 

**Table 1:** General characteristics of the study participants (n = 114)

Characteristics		Frequency	Percent (%)
Gender			
Male		91	79.8
Female		23	20.2
Age			
18-30 years old		23	20.2
31-40 years old		33	28.9
41-50 years old		31	27.2
51-60 years old		19	16.7
>60 years old		8	7.0
BMI			
Overweight	(25-29.9 kg/m^2^)	14	12.4
Obesity class I	(30-34.9 kg/m^2^)	27	23.7
Obesity class II	(35-39.9 kg/m^2^)	36	31.9
Obesity class III	(40-49.9 kg/m^2^)	36	31.9

**Figure 1:**
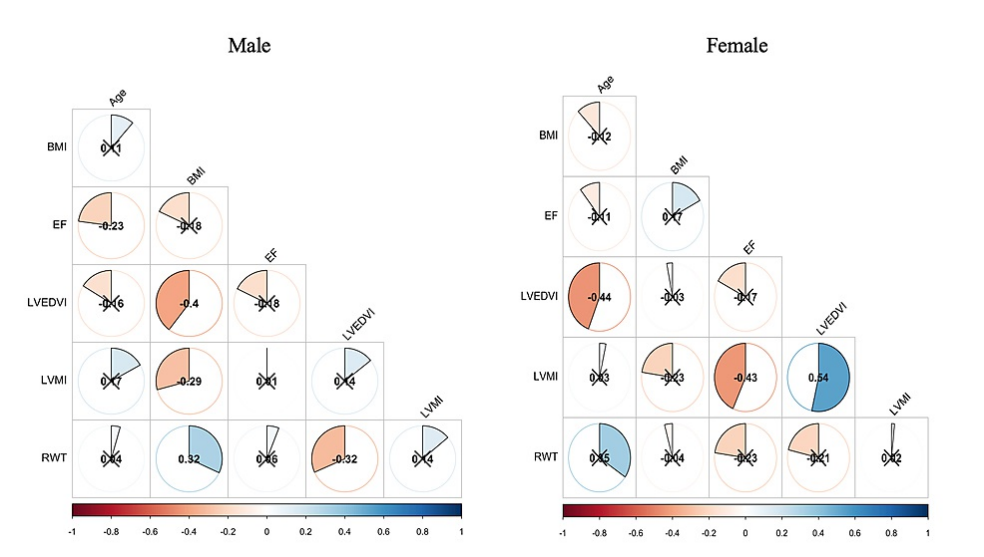
Correlation matrix for male and females Blue indicates a positive correlation and red indicates a negative correlation. X indicates a correlation that is not significant (P > 0.05). BMI: body mass index, BSA: body surface area, EF: ejection fraction, LVEDVI: left ventricular end-diastolic volume index, LVMI: left ventricular mass index, RWT: relative wall thickness.

With regards to morphological and functional properties of the heart, the mean ± SD of LV ejection fraction (55.7% ± 2.8%) was approximately equal between males and female 55.7% vs 55.8%, respectively, and it was found that there is no correlation with the BMI for males and females (male= R 0.093, R2 0.032, P-value 0.093; female= R 0.172, R2 0.029, P-value 0.434). The mean ± SD of left ventricular mass index normally is (28.5±5.84 g/m^2^), it was higher in males than females (28.9 vs 27 g/m^2^) but was not statistically significant (p-value= 0.2). The LVMI was found to have a negative correlation between BMI and male gender that was significant (R - 0.292, R2 0.09, P-value 0.0052), however, there was no correlation with the BMI for females (R - 0.226, R2 0.05, P-value 0.37). The mean ± SD of LVEDI was (48.3±11.6 ml/m^2^) and it was higher in males than females (48.8 vs 46.4 ml/m^2^) but was not statistically significant (p-value= 0.395). There was a negative correlation between BMI and LVEDI in male gender (male= R - 0.396, R2 0.157, P-value 0.0001) while not significant in females (R - 0.0298, R20.0009, P-value 0.893). The mean ± SD of relative wall thickness (RWT) was (0.378±0.07) and it was slightly lower in males than females (0.374 vs 0.394) but was not statistically significant (p-value= 0.149). There was a positive correlation between BMI and RWT in male gender (male= R 0.32, R2 0.12, P-value 0.002) while not significant in females (R - 0.04, R2 0.002, P-value 0.851; Table 2 and Figure 1).

**Table 2:** Morphological and functional properties of the heart and characteristics of study participants stratified by gender BMI: body mass index, BSA: body surface area, EF: ejection fraction, LVEDVI: left ventricular end-diastolic volume index, LVMI: left ventricular mass index, RWT: relative wall thickness.

Heart properties	Overall (mean ± SD)	Gender		P-value
		Male	Female	
Age	41.3±12.4	39.6	47.8	0.007*
BMI (kg/m^2^)	39±9.3	37.4	45.1	0.003*
BSA (m^2^)	2.32±0.16	2.33	2.27	0.158
EF (%)	55.7±2.8	55.7	55.8	0.894
RWT	0.378±0.07	0.374	0.394	0.149
LVEDVI (ml/m^2^)	48.3±11.6	48.8	46.4	0.395
LVMI (g/m^2^)	28.5±5.84	28.9	27	0.2

## Discussion

There are many conflicts in the literature regarding the effects of obesity on the heart. Iacobellis et al. showed that there is no association between BMI and LVH or systolic function [13]. However, they found that there is a significant association only with the impairment of diastolic function [13]. Russo et al., had similar results, where he found that the BMI was associated with worse LV diastolic function independent of LV mass and associated risk factors [14]. On the other hand, Turkbey et al., in a large multiethnic cohort study, concluded that there was a significant correlation between obesity and concentric LV remodeling with no changes in the ejection fraction [15]. All associations were stronger for men than for women. Tumuklu et al. agreed with him and found that obesity is associated with morphologic alterations in the left ventricle and left atrium with subclinical changes in left ventricular systolic function [16]. Moreover, Son et al. reported that an increase in waist circumference was significantly associated with a progressive increase in LA volume index, LV end-diastolic and end-systolic dimensions, and LVMI [17]. Several studies have demonstrated increased LA dimensions in general obesity compared with normal-weight subjects [18-20].

Alpert et al. reported similar results where he found that the more years the patient is obese the higher association with higher LVM, poorer LV systolic function, and greater impairment of LV diastolic filling [4]. They also reported that weight loss induces a decrease in LVM and improvements in LV systolic function and diastolic filling are due in part to favorable alterations in LV loading conditions. Tsioufis et al., however, found that central obesity was associated with diastolic dysfunction only in females [21]. 

We have not found similar results. This could be because of the different populations and ethnicities [22]. The difference could be because some studies included patients who had co-morbidities like, hypertension, diabetes, dyslipidemia, etc. Also, some studies did not separate the male and females. This is necessary due to the fact that it is well known that males have a higher left ventricular mass. Obesity-induced cardiomyopathy is a new and unclear entity. Several theories have been proposed to rationalize the development of obesity-induced cardiomyopathy, but what is well known is that there is a clear relationship between the duration of morbid obesity and the development of cardiomyopathy [23].

Limitations of this study included the lack of a longitudinal approach by which obese individuals could be followed until the incidence of morphological and functional changes of the heart. In addition, if a control group of normal-weight persons was compared to the obese group, the effect of obesity on heart morphology or function would be more obvious. Lastly, the small sample size especially in females significantly will affect the generalizability of our data.

## Conclusions

In conclusion, we have found that the function of the heart is not affected by isolated obesity, as we have found no correlation between the BMI with the ejection fraction. However, indexed cardiac structures like left ventricular mass and left ventricular end-diastolic volume were negatively correlated with higher BMI and positively correlated with RWT only in males. This negative correlation might be one of the triggers to the development of obesity-induced cardiomyopathy.

## References

[1] Sioco G (1998). The heart and lung in obesity. Tex Heart Inst J.

[2] Smith HL, Willius FA (1933). Adiposity of the heart: a clinical and pathologic study of one hundred and thirty-six obese patients. Arch Intern Med.

[3] Leopold JA (2015). Obesity-related cardiomyopathy is an adipocyte-mediated paracrine disease. Trends Cardiovasc Med.

[4] Alpert MA, Omran J, Mehra A, Ardhanari S (2014). Impact of obesity and weight loss on cardiac performance and morphology in adults. Prog Cardiovasc Dis.

[5] Olivotto I, Maron BJ, Tomberli B (2013). Obesity and its association to phenotype and clinical course in hypertrophic cardiomyopathy. J Am Coll Cardiol.

[6] Ng M, Fleming T, Robinson M (2014). Global, regional, and national prevalence of overweight and obesity in children and adults during 1980-2013: a systematic analysis for the Global Burden of Disease Study 2013. Lancet.

[7] Al-Quwaidhi AJ, Pearce MS, Critchley JA, Sobngwi E, O'Flaherty M (2014). Trends and future projections of the prevalence of adult obesity in Saudi Arabia, 1992-2022. East Mediterr Health J.

[8] Iacobellis G, Ribaudo MC, Leto G, Zappaterreno A, Vecci E, Di Mario U, Leonetti F (2002). Influence of excess fat on cardiac morphology and function: study in uncomplicated obesity. Obes Res.

[9] Lavie CJ, McAuley PA, Church TS, Milani RV, Blair SN (2014). Obesity and cardiovascular diseases: implications regarding fitness, fatness, and severity in the obesity paradox. J Am Coll Cardiol.

[10] Poirier P, Alpert MA, Fleisher LA (2009). Cardiovascular evaluation and management of severely obese patients undergoing surgery: a science advisory from the American Heart Association. Circulation.

[11] Chow E, Bernjak A, Williams S (2014). Risk of cardiac arrhythmias during hypoglycemia in patients with type 2 diabetes and cardiovascular risk. Diabetes.

[12] Peterson LR, Waggoner AD, Schechtman KB, Meyer T, Gropler RJ, Barzilai B, Dávila-Román VG (2004). Alterations in left ventricular structure and function in young healthy obese women: assessment by echocardiography and tissue Doppler imaging. J Am Coll Cardiol.

[13] Iacobellis G, Ribaudo MC, Zappaterreno A, Iannucci CV, Di Mario U, Leonetti F (2004). Adapted changes in left ventricular structure and function in severe uncomplicated obesity. Obes Res.

[14] Russo C, Jin Z, Homma S, Rundek T, Elkind MS, Sacco RL, Di Tullio MR (2011). Effect of obesity and overweight on left ventricular diastolic function: a community-based study in an elderly cohort. J Am Coll Cardiol.

[15] Turkbey EB, McClelland RL, Kronmal RA (2010). The impact of obesity on the left ventricle: the Multi-Ethnic Study of Atherosclerosis (MESA). JACC Cardiovasc Imaging.

[16] Tumuklu MM, Etikan I, Kisacik B, Kayikcioglu M (2007). Effect of obesity on left ventricular structure and myocardial systolic function: assesment by tissue Doppler imaging and strain/strain rate imaging. Echocardiography.

[17] Son J-W, Sung JK, Lee J-W (2016). Abdominal obesity and structure and function of the heart in healthy male Koreans: the ARIRANG study. Medicine.

[18] Ito K, Date T, Kawai M (2011). Morphological change of left atrium in obese individuals. Int J Cardiol.

[19] Stritzke J, Markus MR, Duderstadt S (2009). The aging process of the heart: obesity is the main risk factor for left atrial enlargement during aging the MONICA/KORA (monitoring of trends and determinations in cardiovascular disease/cooperative research in the region of Augsburg) study. J Am Coll Cardiol.

[20] Kizer JR, Bella JN, Palmieri V (2006). Left atrial diameter as an independent predictor of first clinical cardiovascular events in middle-aged and elderly adults: the Strong Heart Study (SHS). Am Heart J.

[21] Tsioufis CP, Tsiachris DL, Selima MN (2008). Impact of waist circumference on cardiac phenotype in hypertensives according to gender. Obesity.

[22] Basavarajaiah S, Boraita A, Whyte G, Wilson M, Carby L, Shah A, Sharma S (2008). Ethnic differences in left ventricular remodeling in highly-trained athletes: relevance to differentiating physiologic left ventricular hypertrophy from hypertrophic cardiomyopathy. J Am Coll Cardiol.

[23] Alpert MA (2001). Obesity cardiomyopathy: pathophysiology and evolution of the clinical syndrome. Am J Med Sci.

